# Response of broiler chickens fed low-energy diets to increase levels of lysolecithin: linking growth performance with lipid metabolism gene expression, gut microbial modulation, intestinal morphology, and economic efficiency

**DOI:** 10.3389/fvets.2026.1921649

**Published:** 2026-10-07

**Authors:** Hanan S. Al-Khalaifah, Wafaa A. Eleraky, Sabry A. A. El-Sayed, Basant M. Shafik, Asmaa T. Y. Kishawy, Doaa Ibrahim, Shimaa A. E. Atwa, Sherief M. Abdel-Raheem, Marwa El-Shahat, Khalid Youssif, Anaam E. Omar

**Affiliations:** 1 Environment and Life Sciences Research Center, Kuwait Institute for Scientific Research, Kuwait City, Kuwait; 2 Department of Nutrition and Clinical Nutrition, Faculty of Veterinary Medicine, Zagazig University, Zagazig, Egypt; 3 Department of Animal Wealth Development, Animal and Poultry Production, Faculty of Veterinary Medicine, Benha University, Toukh, Egypt; 4 Department of Biochemistry and Molecular Biology, Faculty of Veterinary Medicine, Benha University, Toukh, Egypt; 5 Department of Public Health, College of Veterinary Medicine, King Faisal University, Al-Ahsa, Al Hofuf, Saudi Arabia; 6 Veterinary Economic and Farm Management, Department of Animal Wealth Development, Faculty of Veterinary Medicine, Zagazig University, Zagazig, Egypt

**Keywords:** beneficial microbes, broilers, duodenal morphology, hydrolyzed lecithin, lipid metabolism, performance

## Abstract

Lowering dietary energy density cuts feed costs but impairs broiler growth due to the limited fat digestion capacity of young birds. Supplementing low energy diets with lysolecithin (a hydrolyzed soy lecithin product) has been reported to counteract this limitation, as its emulsifying action may enhance fat digestion and energy utilization and thereby help to restore performance. This study is designed to evaluate the effects of lysolecithin supplementation on growth performance, remodeling of lipid genes metabolism and intestinal microbiota, antioxidant status, immunological response, and intestinal morphology in broiler chickens fed low-energy diets. A total of three hundred twenty (320) broiler chicks (Ross 308 breed) were obtained from a commercial hatchery and randomly allocated to four nutrition regimens (8 replicates/group, 10 chicks/replicate): Group 1, control basal diet with standard energy and no emulsifier (CS); Group 2, control low-energy diet with 80 kcal/kg lower metabolizable energy and no emulsifier (CLow); and Groups 3 and 4, control low-energy diets supplemented with lysolecithin at 125 and 250 mg/kg diet, respectively (CLowE125 and CLowE250). Lysolecithin-supplemented groups significantly outperformed the unsupplemented low-energy group in terms of total body weight, final body weight gain, and feed conversion ratio (*p* < 0.01) without having an impact on feed intake. Serum triglycerides were decreased in the lysolecithin-treated groups compared with the standard-energy group, whereas serum cholesterol was reduced in the low-energy and 125 mg/kg lysolecithin groups. Dietary lysolecithin supplementation dose-dependently remodeled the expression of key intestinal and hepatic lipid transport and metabolism genes (*SLC27A4, PPARα, PPARγ, ACSL1, CD36, NPC1L1, MGAT2, MTTP, APOB,* and *FABP*), with the 250 mg/kg dose eliciting the most pronounced upregulation. Supplementing the low-energy diet with lysolecithin restored the depleted beneficial genera (*Lactobacillus*, *Bifidobacterium*, and *Bacillus* populations) as quantified by real-time quantitative PCR (qPCR). Adding lysolecithin at a level of 250 mg/kg to the low-energy diet improved (*p* < 0.001) oxidative and immune status by lowering malondialdehyde and increasing catalase, superoxide dismutase, lysozyme, total antioxidant capacity, and serum IgM. By increasing villus height and the villus height-to-crypt depth ratio (*p* < 0.001), duodenal morphology was positively impacted by lysolecithin supplementation. Overall, the lysolecithin emulsifier is a promising nutritional approach that enhances feed and economic efficiency while maintaining production standards under energy-restricted diets.

## 1 Introduction

In the poultry industry, lowering production costs without sacrificing product quality is a long-term objective. Poultry production becomes more sustainable and efficient when feed nutrient consumption rises (1–4). Given that fat has the utmost caloric value of all the necessary elements, increasing the fat content of poultry feed is a common practice in modern poultry production to satisfy the increased energy needs of broilers. Adding fat to formulated diets for broiler chickens increases feed costs, and broilers also face difficulties with lipid utilization and digestion. This prevents them from producing and secreting enough bile salts and lipase, which can explain their inadequate lipid absorption and digestion (5). The breakdown of water-insoluble triglycerides is necessary in order to digest and absorb dietary fat. Bile salts emulsify dietary lipids, and pancreatic lipase hydrolyses them (6). Improving fat digestibility is a practical way to cut back on extra lipids in broiler foods, which lowers feed production prices without affecting broiler chicken performance (7, 8). Intestinal lipid absorption is not a passive process but is mediated by a coordinated set of genes expressed in the enterocyte. Long-chain fatty acids are taken up across the brush border by transporters such as CD36 and fatty-acid transport protein 4 (SLC27A4/FATP4) and are trafficked within the cell by fatty-acid-binding proteins (FABP), whereas cholesterol uptake is mediated by NPC1L1 (9). Absorbed lipids are re-esterified by enzymes including acyl-CoA synthetase (ACSL) and monoacylglycerol acyltransferase (MGAT2) and are assembled, together with apolipoprotein B (APOB) and microsomal triglyceride transfer protein (MTTP) into chylomicrons for export; these pathways are under the transcriptional control of peroxisome proliferator-activated receptors (10). Emulsifiers are one type of feed additive that may be applied to broiler diets to increase calories per unit of feed content by improving the digestion as well as absorption of fat (11). Lysolecithin, a lysophospholipid produced by enzymatic hydrolysis of lecithin, is a powerful biosurfactant with strong hydrophilic properties; by forming smaller and more stable micelles, it enhances lipase access to dietary fat and improves lipid digestion and absorption (12). By increasing the luminal supply of absorbable fatty acids and monoacylglycerols, lysolecithin can modulate the expression of these absorption- and transport-related genes. Supplementing broiler diets with lysolecithin or related emulsifiers has been shown to upregulate intestinal fatty-acid-uptake and lipid-metabolism genes and improve fat digestibility (13, 14). However, the dose-dependent effect of lysolecithin on the full panel of intestinal lipid absorption and transport genes in broilers fed a low-energy diet has received limited attention. The fatty compound lecithin, which occurs naturally in a variety of food sources such as whole grains, egg yolks, soybeans, and milk, is the source of lysolecithin (15). However, hydrolyzed lecithins greatly differ from crude lecithins, as lecithin must be treated with Phospholipase A2 under heat and pressure to be hydrolyzed to lysolecithin (16). Phospholipase A2 catalyzes the conversion of soy-lecithin into lysophosphatidylcholine (LPC). Because one fatty acid is removed, it possesses more hydrophilic qualities than ordinary phospholipids (17, 18). Because LPC activity increases a measure of total tract apparent digestibility of fatty acids, LPC supplementation has been linked to higher body weight gain (BWG) in broiler chickens during the starting phase (19). Because the microbiota is shaped by diet, even modest changes in feed formulation can reshape its structure; dietary fat source and concentration, in particular, significantly alter the relative abundance of the dominant caecal phyla, Firmicutes and Bacteroidetes. Lysolecithin can preserve mucosal integrity, promote the development of advantageous microbial communities, and have a good effect on gut health. Lysophospholipid supplementation has been reported to increase the caecal Lactobacillus population and modulate the microbial community of broilers fed reduced-energy diets (20, 21). Beneficial genera such as Lactobacillus, Bifidobacterium, and Bacillus support gut barrier function and colonization resistance (22). In broiler chickens, lysolecithin may also be essential for preserving intestine architecture and health. At 28 days, lysolecithin administration resulted in changes to the duodenum’s shape and a decrease in mucosal thickness (23). All of these factors work together to increase chicken performance, feed efficacy, and general wellbeing (24–26).

Nevertheless, how lysolecithin affects the broiler intestinal microbiota when dietary energy is restricted remains insufficiently characterized. The present study therefore examined the bacterial community of broiler chickens fed a low-energy diet with or without graded levels of lysolecithin. To the best of our knowledge, no study has yet provided an integrated, dose–response evaluation linking the molecular, microbial, structural and physiological responses to lysolecithin within a single low-energy-diet model. The present study was therefore designed to address this gap by using a broiler chicken model to comprehensively assess the influence of graded levels of lysolecithin, supplemented as an exogenous emulsifier in a low-energy diet, on (i) growth performance and carcass traits, (ii) the expression of intestinal genes governing lipid absorption and transport, (iii) gut bacterial load, (iv) intestinal morphology, and (v) blood biochemical indices, antioxidant capacity, and serum immunoglobulin concentrations. We hypothesized that supplementing a low-energy (−80 kcal ME/kg) broiler diet with graded levels of a hydrolyzed soy lecithin product would counteract the growth penalty of energy restriction in a dose-dependent manner by enhancing intestinal lipid digestion and absorption, up-regulating lipid-transport and -metabolism genes, improving intestinal morphology, and restoring beneficial gut microbiota, thereby recovering growth performance, health status, and economic efficiency to levels comparable with a standard-energy diet.

## 2 Materials and methods

### 2.1 Ethical approval

The Poultry Research Unit at Zagazig University’s Faculty of Veterinary Medicine in Egypt served as the site of the experimental trial. Every experiment’s protocol was authorized by the Institutional Animal Care and Use Committee (ZU-IACUC) of Zagazig University, Egypt (Approval No. ZU-IACUC/2/F/199/2025).

### 2.2 Feed additive used

The feed additive used was Lecipro LeciOIL™ Hy50, produced by Orison Chemicals Limited (Yihang Int’l Building No.5, Binhai New Area, Tianjin, China). It is a highly enzymatically hydrolyzed soy lecithin liquid product with an elevated lysophospholipid content, purpose-designed for applications that demand effective emulsification, superior dispersibility, and enhanced interfacial performance. Its production involves a carefully controlled enzymatic hydrolysis process, through which a significant portion of the native phospholipid is transformed into lysophospholipid species including Lys phosphatidylcholine (LPC), Lysophosphatidylethanolamine (LPE), Lysophosphatidic Acid (LPA), and Lysophosphatidylinositol (LPI). Its total phospholipid content is more than 30%, Total Lysophospholipid (LPL) more than 15%, and Lys phosphatidylcholine (LPC) more than 7%.

### 2.3 Birds, experimental design, and feeding program

A total of 320 one-day-old male Ross 308 broiler chickens were acquired from a commercial hatchery and used in a 35-day feeding trial, with an initial average body weight of 45.52 ± 2 g. The chicks were grown on appropriate litter in a well-ventilated environment. The temperature in the room was kept at 34 °C for the first week before being progressively lowered until it reached 25 °C at the conclusion of the rearing stage. For the first week, the lighting program ran twenty-four hours a day. Over the course of seven to thirty-five days, the lighting scheme was modified from 24 h of light each day to 20 h of light and 4 h of darkness. Birds were reared in floor pens each measuring 1.0 × 1.0 m (1 m^2^ housing 10 birds), in accordance with commercial Ross 308 stocking-density recommendations. The birds were nourished ad-libitum and given access to clean water. The current study employed a three-phase nourishing plan with starter (1–10 d), grower (11–23 d), and finisher (24–35 d) diets. Eight replicates of ten broiler chickens were randomly allocated to four treatments (80 birds /group). Group 1 consisted of a control basal diet with standard energy required without emulsifier (CS), Group 2 was a control low-energy group in which decreased nutritional density test food (80Kcal/Kg lower ME) was given without emulsifier (C_Low_), Group 3 consisted of a control low energy diet + lysolecithin at 125 mg /kg (C_Low_E_125_), while Group 4 was a control low energy diet + 250 mg lysolecithin /kg group (C_Low_E_250_). The basal diet was designed to satisfy the needs of developing Ross chicks in accordance with the Ross manual guide published by AVIAGEN (27) as shown in Table 1. All feedstuffs and diets were analyzed for different nutrients (28). The hydrolyzed soy lecithin product was first pre-blended with a small portion of the ground corn-soybean meal, which was then incorporated into the remaining ingredients to ensure uniform distribution throughout the diet which was provided in mash form.

**Table 1:** Proximate and chemical composition of the experimental diets (%).

Ingredients	Starter period (1–10 d)		Grower period (11–23 d)		Finisher period (24–35 d)	
	Normal ME	Low ME	Normal ME	Low ME	Normal ME	Low ME
Yellow corn	49	49.2	49.8	51	55.1	55.95
Soybean meal, 48%	40.13	39.9	36.9	36.42	31.25	30.9
Wheat Bran	3	4.05	4.8	5.4	5.6	6.4
Soybean oil	4	2.93	5.16	3.86	5.26	4
Calcium carbonate	1.1	1.15	1	1	0.9	0.9
Calcium dibasic phosphate	1.52	1.48	1.2	1.2	0.8	0.8
Common salt	0.3	0.3	0.3	0.3	0.3	0.3
Premix *	0.3	0.3	0.3	0.3	0.3	0.3
DL-methionine, 98%	0.14	0.12	0.16	0.14	0.1	0.08
Lysine HCl, 78%	0.14	0.1	0.02	0.01	0.03	0.01
Choline 60%	0.06	0.08	0.07	0.07	0.07	0.07
Threonine	0.05	0.03	0.03	0.02	0.03	0.03
Phytase	0.01	0.01	0.01	0.01	0.01	0.01
Na_2_Co_3_	0.2	0.3	0.2	0.22	0.2	0.2
Anti-mycotoxin	0.05	0.05	0.05	0.05	0.05	0.05
Total	100	100	100	100	100	100
Chemical composition						
ME (Kcal/kg)**	2976.5	2897.3	3049.93	2,971	3104.90	3024.20
Crude protein %	22.84	22.85	21.56	21.50	19.46	19.45
Calcium %	0.925	0.934	0.801	0.801	0.649	0.651
Available phosphorus %	0.509	0.506	0.446	0.448	0.357	0.360
Lysine %	1.568	1.603	1.507	1.528	1.415	1.446
Methionine %	0.525	0.529	0.568	0.562	0.502	0.501
Threonine %	1.048	1.079	1.067	1.084	1.03	1.06

*Premix per kg of diet: Vitamin A, 12,500 IU; Vitamin D_3_, 2,500 IU; Vitamin E, 30 IU; Vitamin K_3_, 2.65 mg; thiamine, 1.8 mg; riboflavin, 3.6 mg; pantothenic acid, 10 mg; folic acid, 0.55 mg; pyridoxine, 3.5 mg; niacin, 35 mg; cobalamin, 0.01 mg; biotin, 0.15 mg; Fe, 80 mg; Cu, 8 mg; Mn, 60 mg; Zn, 40 mg; I, 0.35 mg; Se, 0.15 mg. **computed in accordance with NRC (36). ME, metabolic energy; CP, crude protein; EE, ether extract; CF, crude fiber; Ca, calcium; P, phosphorus.

### 2.4 Growth performance

Each chick was weighed separately to estimate their starting weights, and on days 1, 10, 23, and 35, the average body weight (BW) of every group was measured. The body weight gain (BWG) was calculated using the difference between the initial and final weight at each phase interval (29, 30). The difference between the amount of feed supplied and the amount left over, which was then divided by the total number of birds, was used to determine the average feed intake (FI) per bird in each replicate. The ratio of feed consumption to weight increase in grams was used to express feed conversion (FCR) (31, 32).

### 2.5 Carcass traits

At the end of the experiment, eight birds were randomly selected from each group and fasted overnight with free access to water. The following day, each bird was weighed, humanely slaughtered, scalded at 60–70 °C, defeathered, eviscerated, and washed with tap water. The carcass, with the skin retained, was weighed to determine carcass yield, which was expressed as a percentage of the live body weight. Edible carcass yield was calculated as the combined weight of the carcass, neck, giblets (liver, heart, and gizzard), and legs, expressed as a percentage of the live body weight. The liver, heart, and gizzard were individually excised and weighed.

### 2.6 Serum measurements

On the 35th day, eight birds were randomly selected from each group for blood sample collection from their wing veins (*n* = 8). The samples were placed in a clean, dry tube without anticoagulant, allowed to coagulate at room temperature, then centrifuged for 5 min at 3000 rpm at 4 °C to extract the serum. The collected serum was stored in Eppendorf tubes at −20 °Cuntil use for chemical analysis.

Kits from Nanjing Jiancheng Bioengineering Institute (NJBI, Nanjing, China) were used to measure the following: total cholesterol (TC; Kit A110-1-1), serum triglycerides (TG; Kit A110-1-1 T), high-density lipoprotein cholesterol (HDL-C; Kit A111-1-1), and low-density lipoprotein cholesterol (LDL-C; Kit A113-1-1). For determining the albumin/globulin (A/G) ratio, total protein was measured by Abcam Assay Kit ((ab102536) Abcam / BioVision, Cambridge, UK), and albumin was measured by kits from Nanjing Jiancheng Bioengineering Institute ((NJBI) (A003-1-2), Nanjing, China) and globulin fractions of plasma. Kidney parameters such as creatinine, uric acid, and hepatic enzymes ALT (alanine aminotransferase) and AST (aspartate aminotransferase) were also assessed and analyzed using the matching reagent kit’s instructions (Spinreact Co. Spain).

### 2.7 Immune and antioxidant indices

Serum levels of catalase (CAT) (U/mL) and malondialdehyde (MDA) (nmol/mL) were assessed according to Nanjing Jiancheng Bioengineering Institute (NJBI) (A007-1-1 and A003-1-2, respectively); total antioxidant capacity (TAC) (U/ml) was measured via a commercial assay kit (Sigma-Aldrich, MAK187). Superoxide dismutase (SOD) (U/mL) was measured by a commercial assay kit Abcam SOD Assay Kit (ab65354) Abcam / BioVision, Cambridge, UK. Lysozyme /muramidase ELISA kits were used to measure lysozyme activities (LZM) (ng/mL) (33); immunoglobulin M (IgM) was also measured by ELISA kits from CUSABIO ® (34).

### 2.8 Histological examination

Chicken intestinal samples were taken, well-kept for 24 h in a 10% buffered neutral formalin solution, dehydrated in gradually increasing ethanol (70, 80, 95, 95, and 100%), washed in xylene, and then embedded in paraffin. On a Rotatory Microtom microtome (Leica RM 2155, England), paraffin sections with a thickness of 5 μm were cut longitudinally and transversely. Following preparation, the pieces were regularly stained with hematoxylin and eosin stains and observed under microscope (35).

For the intestinal histomorphometry, absorption surface area (ASA), intestinal crypt depth (CD), intestinal villi length (VL), and intestinal villi width (VW) were measured. To measure these parameters, 50 correctly associated villi and crypts from each part of every intestinal piece were used, and the results were averaged for every bird. Each villi width was measured at the half-height point, and their elevations were measured from tip to base. A light microscope with a full HD microscopic camera and image analysis software (Leica Microsystem, Germany) was used to examine the tissue portions. For statistical analysis, image analysis software was used to measure the parameters. The following design was used to determine the absorption surface area (ASA): ASA (mm2) is equal to villus width x villus height (36).

### 2.9 Assessment of intestinal lipid transport and metabolism gene expression

At 35 days of age, liver and intestinal tissue samples were collected from five birds per experimental group and immediately immersed in TRIzol reagent (Invitrogen Life Technologies, Carlsbad, CA, USA) according to the manufacturer’s protocol for downstream gene expression analysis. The intestinal samples were used to quantify the mRNA abundance of the following: solute carrier family 27 member 4 (*SLC27A4*); cluster of differentiation 36 (*CD36*); Niemann-Pick C1-Like 1 (*NPC1L1*); monoacylglycerol acyltransferase 2 (*MGAT2*); microsomal triglyceride transfer protein (*MTTP*); and Apolipoprotein B (*APOB*) and fatty acid binding protein (*FABP*). Liver samples were used to quantify the following: peroxisome proliferator-activated receptors alpha and gamma (*PPARα, PPARγ*) and long-chain acyl-CoA synthetase (*ACSL*). Total RNA was subsequently isolated using the QIAamp RNeasy Mini Kit (Qiagen, Germany), and RNA concentration and purity were assessed spectrophotometrically prior to downstream applications. Quantitative reverse transcription PCR (RT-qPCR) was conducted in a single-step format on a Stratagene MX3005P real-time thermocycler (Agilent Technologies) using the QuantiTect SYBR Green RT-PCR Kit (Qiagen Germany) in accordance with the manufacturer’s instructions. Amplification specificity for each target was verified through post-run dissociation (melting) curve analysis to confirm the absence of non-specific products or primer dimers. Glyceraldehyde-3-phosphate dehydrogenase (*GAPDH*) served as the endogenous reference gene for normalization of transcript levels across all samples. Primer sequences for each target gene are provided in Table 2. Relative mRNA expression was calculated using the 2^−ΔΔCt method, with data expressed as fold-change relative to the reference group (37).

**Table 2:** Primers’ sequences that are employed for quantitative reverse transcription polymerase chain reaction assays.

Specificity/Target gene	Primer sequence (5′-3′)	Accession No./Ref
Fatty acids transportation and metabolism-related genes		
*SLC27A4*	F- TTCTACCTGGGATCAGGGGG R- AAGTGCAGACTCAGGGCTTC	XM_015279553.1
*PPARα*	F- GAATCACCCAGTGGAGCAGAAAA R- TGGCAGCAGTGGAAGATTCG	AF163809.1
*PPARγ*	F-CTCCGGCTGAGACTTGACAG R-AATTAACGGGCCAAAACGGC	NM_001001460.2
*ACSL1*	F- AGCCTGGGAGCATTTGTGTT R- CCCAGTGCTGGAACCTGAAT	XM_015276227.4
*CD36*	F: AATGACACCATCACGTGCCT R: CGAGGAACAGCACAGAACCT	XM_015301527.4
*NPC1L1*	F- CAACTTCGCCATCACTGTGC R- ACTGCGGTGTGTCCTCAAAG	XM_040657257.2
*MGAT2*	F- TCTTTCACACGGGAGACTGC R- ATCGTCTCGGGGAACAGGTA	XM_015276736.4
*MTTP*	F- CACGTACGGCCTAGACATCC R- CTGTGAAAACTGCACCGTGG	NM_001109784.3
*APOB*	F- TCTACTCTCCAGGTCCCAGC R- AACGTCAGCAAATGTTGGGC	NM_001044633.2
*FABP*	F- CTTTGCCCCCAGACTCTCAC R- ACCCATACCACCTACGACCA	AY648562.1
Bacterial counting genes		
*Bacteroidetes*	F: GGARCATGTGGTTTAATTCGATGAT R: AGCTGACGACAACCATGCAG	(86)
*Bifidobacterium species*	F: TCGCGTCYGGTGTGAAAG R: CCACATCCAGCRTCCAC	(87)
*Firmicutes*	F: GGAGYATGTGGTTTAATTCGAAGCA R: AGCTGACGACAACCATGCAC	(86)
*Bacillus* spp.	F: GGGAAACCGGGGCTAATACCG R: CTTCCTCCGTTTTGTCAACAC	(88)
*Enterobacteriaceae*	F: CATTGACGTTACCCGCAGAAGAAGC R: CTCTACGAGACTCAAGCTTGC	(89)
*Lactobacillus species*	F: AGCAGTAGGGAATCTTCCA R: CACCGCTACACATGGAG	(90)
House keeping		
GAPDH	F-GGTGGTGCTAAGCGTGTTA R-CCCTCCACAATGCCAA	NM205518
*TBP*	F-GTCCACGGTGAATCTTGGTT R-GCGCAGTAGTACGTGGTTCTC	Acc:8484
*rpoD*	F-ACATCGCTAAACGTATCGAA R-GTACTGTTCCAGCAGATAGG	(91)

SLC27A4, Solute carrier family 27 member 4; PPARα, PPARγ, peroxisome proliferator-activated receptors alpha and gamma; ACSL1, long-chain acyl-CoA synthetase family member 1; CD36, cluster of differentiation 36; NPC1L1, Niemann-Pick C1-Like 1; MGAT2, monoacylglycerol acyltransferase 2; MTTP, microsomal triglyceride transfer protein; APOB, apolipoprotein B; FABP, fatty acid binding protein; GAPDH, Glyceraldehyde-3-phosphate dehydrogenase; TBP, TATA-binding protein; rpoD, RNA polymerase sigma factor.

### 2.10 Quantification of bacterial load via DNA copy number analysis

To assess microbial populations, cecal contents were collected at 35 days of age. These samples were used to quantify the DNA copies of key bacterial groups, including *Enterobacteriaceae, Firmicutes*, *Lactobacillus*, *Bacteroides*, *Bacillus* spp., and *Bifidobacterium*. Genomic DNA was isolated from all collected samples using the QIAamp DNA Stool Mini Kit (Qiagen, Germany), strictly adhering to the manufacturer’s recommended protocol. The purity and concentration of the extracted DNA were assessed spectrophotometrically using a NanoDrop 2000 instrument (Thermo Fisher Scientific, Waltham, MA). All purified DNA samples were stored at −80 °C until further analysis. Quantitative PCR (qPCR) was subsequently performed to enumerate *Enterobacteriaceae, Firmicutes*, *Lactobacillus*, *Bacteroides*, *Bacillus* spp., and *Bifidobacterium* using a Stratagene MX3005P thermocycler in 96-well polypropylene plates. Each reaction was conducted in triplicate in a total volume of 25 μL, consisting of 12.5 μL of 2× QuantiTect Probe RT-PCR Master Mix (Qiagen, Germany), 0.25 μL of each primer (50 pmol), 0.125 μL of FAM-TAMRA labeled probe (30 pmol), 3 μL of DNA template, and 8.875 μL of PCR-grade water. Primer sequences used for the detection of all investigated bacterial taxa are listed in Table 2. For absolute quantification, standard calibration curves were constructed using 10-fold serial dilutions of DNA extracted from pure bacterial cultures and mixed cecal microbial communities. These dilutions were subjected to qPCR to establish the relationship between threshold cycle (Ct) values and known DNA concentrations. Bacterial counts for cecal microbiota were subsequently determined by interpolating sample Ct values against the respective standard curves, with final results expressed as log₁₀ CFU/g of sample.

### 2.11 Economic efficiency

The economic efficiency of the dietary treatments was evaluated on a per-bird basis over the whole experimental period (0–35 d) (38). The total feed cost was calculated by multiplying the total feed intake (kg) of each bird by the price of one kilogram of the corresponding diet, with prices of 0.410, 0.400, 0.4013, and 0.4025 US$/kg for the CS, C_Low_, C_Low_E_125_, and C_Low_E_250_ diets, respectively. A fixed cost of 0.030 US$/birds (representing non-feed expenses such as chicks, labor, medication, and management, assumed equal across all groups) was added to the total feed cost to obtain the total cost. The total revenue was calculated as the final body weight (kg) of each bird multiplied by the selling price of one kilogram of live body weight (1.50 US$/kg). The economic indices were then computed using the following equations:

- Feed cost per kilogram gain (US$) = total feed cost ÷ total body weight gain (kg)
- Total feed cost (US$) = total feed intake (kg) × price of 1 kg diet
- Total cost (US$) = total feed cost + fixed cost
- Total revenue (US$) = final body weight (kg) × selling price of 1 kg live weight
- Net revenue (US$) = total revenue − total cost
- Economic efficiency (EEf) = net revenue ÷ total cost
- Profitability index = net revenue ÷ total revenue
- Benefit–cost ratio (BCR) = total revenue ÷ total cost

All monetary values were expressed in US dollars (US$) at the prevailing market prices during the experimental period.

### 2.12 Statistical analysis

All collected experimental data were subjected to statistical evaluation using the General Linear Model (GLM) procedure within SPSS software. Prior to analysis, the homogeneity of variance across all treatment groups was verified using Levene’s test, while the normality of data distribution was confirmed through the Shapiro–Wilk test. Where statistically significant differences were detected, Tukey’s post-hoc test was applied for multiple comparisons between group means. Results values are stated as ±standard error (SE), and a probability threshold of *p* < 0.05 was adopted as the criterion for statistical significance. All figures and graphical representations were constructed using GraphPad Prism (Version 8). Orthogonal polynomial contrasts (linear and quadratic) describing the response to lysolecithin were computed only across the three low-energy dietary treatments in ascending order of lysolecithin inclusion (CLow = 0, CLowE125 = 125, and CLowE250 = 250 mg/kg); the standard-energy control (CS) served solely as a reference group.

## 3 Results

### 3.1 Growth performance

The effects of adding lysolecithin emulsifier at two levels (125 and 250 mg/kg diet) to a low-energy diet (−80 kcal ME/kg) on broiler performance are presented in Table 3. Initial body weight did not differ between groups (*p*>0.05), which indicates homogenous groups at the start of trial. During the starter period (1–10 days), BW (*p*=0.001) and BWG (*p*=0.001) were significantly increased, while feed intake (*p*=0.004) decreased, in chicks fed the standard basal diet or the low-energy diet supplemented with a high level of lysolecithin, compared with those fed the low-energy diet without emulsifier or with a low level of emulsifier. However, feed conversion ratio (*p*=0.001) was significantly improved in all treatment groups compared with those fed the low-energy diet without emulsifier. During the grower period (11–23 day), BW (*p*<0.001) and BWG (*p*=0.001) increased in broilers fed a basal diet and a low-energy diet supplemented with high levels of lysolecithin compared to those fed on a low-energy diet without emulsifiers and those fed the 125 mg/kg diet lysolecithin. FI was high (*p*=0.010) in group 3, which had a low-energy diet with 125 mg /kg of lysolecithin, compared with other groups. The best FCR was detected (*p*<0.001) in group 1, which received a diet with standard energy, and group 4, which received lysolecithin at a rate of 250 mg/kg diet.

**Table 3:** Effect of the supplementation of a low-energy diet with lysolecithin on the growth performance of broilers in different growth stages.

Traits	CS	C_Low_	C_Low_E_125_	C_Low_E_250_	ANOVA value	Regression	
						Linear	Quadratic
Initial BW (g)	46.81 ± 0.09	45.15 ± 1.22	44.80 ± 1.22	45.33 ± 2.19	0.367	0.222	0.210
Starter period (1–10th day)							
BW (g)	326.87 ± 5.33^a^	310.40 ± 1.10^b^	317.73 ± 4.12^b^	326.33 ± 2.73^a^	0.001	0.521	<0.001
BWG (g)	280.06 ± 4.38^a^	265.25 ± 1.74^c^	272.93 ± 4.57^b^	281.00 ± 0.60^a^	0.001	0.252	0.001
FI (g)	347.27 ± 1.79^b^	363.39 ± 1.32^a^	357.54 ± 2.89^a^	348.44 ± 1.53^b^	0.004	0.725	0.001
FCR	1.24 ± 0.02^c^	1.37 ± 0.01^a^	1.31 ± 0.04^b^	1.24 ± 0.01^c^	0.001	0.427	<0.001
Grower period (11th–23rd day)							
BW (g)	1,209 ± 13.89^a^	1114.13 ± 5.52^b^	1141.87 ± 8.95^b^	1198.47 ± 25.87^a^	<0.001	0.926	<0.001
BWG (g)	882.13 ± 11.55^a^	803.73 ± 5.94^b^	824.13 ± 12.93^b^	872.13 ± 27.53^a^	0.001	0.828	<0.001
FI (g)	1194.7 ± 14.11^b^	1190.71 ± 4.35^b^	1236.67 ± 15.28^a^	1204.67 ± 15.01^b^	0.010	0.034	0.099
FCR	1.36±0.02^b^	1.48 ± 0.02^a^	1.50 ± 0.01^a^	1.38 ± 0.03^b^	<0.001	0.127	<0.001
Finisher period (24–35th day)							
BW (g)	2,248 ± 38.63^a^	2111.7 ± 2.52^b^	2112.3 ± 11.15^b^	2247.3 ± 16.65^a^	<0.001	0.982	<0.001
BWG (g)	1,039 ± 25.51^a^	997.53 ± 4.22^b^	970.47 ± 10.80^b^	1048.87 ± 19.07^a^	0.002	0.955	<0.001
FI (g)	1523.9 ± 43.82	1505.57 ± 30.95	1546.7 ± 57.74	1490.7 ± 10.07	0.400	0.584	0.435
FCR	1.47 ± 0.06^bc^	1.51 ± 0.04^b^	1.59 ± 0.05^a^	1.42 ± 0.02^c^	0.008	0.627	0.003
Overall performance (1–35th day)							
BW (g)	2,248 ± 38.63^a^	2111.7 ± 2.52^b^	2112.3 ± 11.15^b^	2247.3 ± 16.65^a^	<0.001	0.982	<0.001
BWG (g)	2201.19 ± 38.6^a^	2066.55 ± 2.44^b^	2067.50 ± 9.94^b^	2201.97 ± 18.79^a^	<0.001	0.953	<0.001
FI (g)	3065.87 ± 57.01	3060.21 ± 25.32	3140.91 ± 58.95	3043.81 ± 14.79	0.104	0.868	0.158
FCR	1.39 ± 0.04^b^	1.48 ± 0.02^a^	1.52 ± 0.02^a^	1.38 ± 0.01^b^	<0.001	0.843	<0.001

^a–c^Means within the same row with different superscripts are significantly different at *p* < 0.05.

CS, control standard energy diet without emulsifier; C_Low_, control low-energy diet without emulsifier; C_Low_E_125_, control low-energy diet + 125 mg/kg diet lysolecithin; C_Low_E_250_, control low-energy diet+250 mg/kg diet lysolecithin. BW, body weight; BWG, body weight gain; FI, Feed intake; FCR, feed conversion ratio.

Throughout the finisher period (24–35 days), birds in groups 1 and 4, which were fed a control standard diet and low-energy diet with high levels of emulsifier, respectively, had higher BW (*p*<0.001) and BWG (*p*=0.002) than the other groups. FI displayed no significant variances (*p*=0.400). The best FCR (*p*=0.008) was detected in group 4, which fed on high level of lysolecithin. During the overall period, BW and BWG showed an improvement (*p*<0.001) in group 4 fed on high emulsifier level than group 2 fed on energy restricted diet without emulsifier and restore their level to be comparable with group 1 which fed basal diet. FI was not affected in all treatment groups. The overall FCR improved (*p*<0.001) in groups 1 and 4 compared with group 2 and 3. The supplementation of low-energy diets with lysolecithin at 250 mg/kg diet restored BW, BWG, and FCR to values statistically similar to group 1 which receive control standard energy diet with no effect on total feed intake.

### 3.2 Carcass traits

Table 4 shows the influence of including a lysolecithin emulsifier in low-energy diets on carcass characteristics. There was no noteworthy changes (*p*>0.05) in dressing proportion observed among groups. There was a linear increase in gizzard percentage (*p*=0.030) in supplemented groups with emulsifiers. Liver percentage was unaffected by treatment (*p*>0.05).

**Table 4:** Effect of the dietary supplementation of lysolecithin to a low-energy diet on carcass traits of broilers.

Traits	CS	C_Low_	C_Low_E_125_	C_Low_E_250_	ANOVA value	Regression	
						Linear	Quadratic
Dressing %	77.17 ± 1.04	76.08 ± 2.14	75.90 ± 1.47	77.57 ± 1.47	0.658	0.841	0.243
Liver %	3.07 ± 0.27	2.62 ± 0.27	2.60 ± 0.09	2.53 ± 0.07	0.148	0.073	0.230
Heart %	0.47 ± 0.06	0.35 ± 0.02	0.50 ± 0.12	0.46 ± 0.04	0.118	0.453	0.380
Gizzard %	2.07 ± 0.19	2.33 ± 0.17	2.46 ± 0.33	2.46 ± 0.08	0.116	0.030	0.444

CS, control standard energy diet without emulsifier; C_Low_, control low-energy diet without emulsifier; C_Low_E_125_, control low-energy diet + 125 mg/kg diet lysolecithin; C_Low_E_250_, control low-energy diet + 250 mg/kg diet lysolecithin.

### 3.3 Blood biochemical parameters

Lysolecithin’s impact on the blood biochemical indices of broilers is shown in Table 5. ALT activity was significantly (*p*=0.020) reduced in groups 2, 3 and 4 compared with group 1, while supplementation of low energy diet with lysolecithin at 250 mg/kg diet partially restore ALT value indicating improved hepatic function. In contrast, AST remained unaffected (*p*>0.05).

**Table 5:** Effect of the dietary supplementation of lysolecithin to a low-energy diet on the biochemical blood parameters of broilers.

Traits	CS	C_Low_	C_Low_E_125_	C_Low_E_250_	ANOVA value	Regression	
						Linear	Quadratic
Liver function tests							
ALT (U/L)	9.00 ± 1.73^a^	5.67 ± 0.58^b^	5.67 ± 0.58^b^	6.67 ± 1.15^b^	0.020	0.042	0.010
AST (U/L)	204 ± 47.31	174.33 ± 8.96	169.3 ± 10.21	232.3 ± 128.39	0.665	0.676	0.272
Kidney function tests							
Creatinine (mg/dl)	0.23 ± 0.03^c^	0.44 ± 0.08^a^	0.28 ± 0.03^bc^	0.33 ± 0.04^b^	0.005	0.295	0.030
UA (mg/dl)	2.50 ± 0.25^c^	11.01 ± 1.08^a^	6.43 ± 0.79^b^	3.17 ± 0.32^c^	<0.001	0.194	<0.001
Protein profile							
Albumin (g/dl)	1.54 ± 0.06	1.41 ± 0.05	1.57 ± 0.11	1.47 ± 0.17	0.317	0.870	0.876
Total protein (g/dl)	3.5 ± 0.01	3.23 ± 0.11	3.67 ± 0.55	3.27 ± 0.32	0.371	0.758	0.731
Globulin (g/dl)	1.92 ± 0.03	1.79 ± 0.60	2.09 ± 0.46	1.80 ± 0.19	0.460	0.940	0.571
A/G ratio	0.79 ± 0.02	0.77 ± 0.02	0.77 ± 0.12	0.81 ± 0.08	0.883	0.697	0.523
Lipid profile							
Cholesterol (mg/dl)	125.67 ± 0.58^a^	97.33 ± 20.53^b^	97.67 ± 3.21^b^	121.3 ± 10.26^ab^	0.029	0.683	0.005
TG (mg/dl)	110.67±14.3^a^	99.00 ± 5.57^b^	103.67 ± 10.1^b^	96.3 ± 5.51^b^	<0.001	0.001	0.004
HDL (mg/dl)	63.00 ± 8.18^b^	61.00 ± 10.58^b^	66.3 ± 6.35^b^	83.3 ± 10.02^a^	0.025	0.021	0.103
LDL (mg/dl)	15.73 ± 12.63	20.20 ± 9.36	18.60 ± 8.32	21.07 ± 6.02	0.401	0.860	0.482

^a–c^Mean values in the same row with different superscripts differ significantly (*p*<0.05).

CS, control standard energy diet without emulsifier; C_Low_, control low-energy diet without emulsifier; C_Low_E_125_, control low-energy diet + 125 mg/kg diet lysolecithin; C_Low_E_250_, control low-energy diet + 250 mg/kg diet lysolecithin; ALT, Alanine aminotransferase; AST, Aspartate aminotransferase; A/G, Albumin/ Globulin ratio; TG, Triglycerides; HDL, High-density lipoprotein; LDL, Low-density lipoprotein; UA, Uric acid.

Renal function markers showed significant differences: creatinine and uric acid (UA) levels increased more in the group fed the low-energy diet without lysolecithin and decreased in the groups fed low-energy diets supplemented with different levels of emulsifier compared with the low-energy diet without emulsifier. The lowest creatinine and UA levels were recorded in the CS group, which was fed the standard-energy diet, followed by group 4, which was fed the low-energy diet with the highest level of lysolecithin.

Protein metabolism indices (Albumin, Total protein, Globulin, and A/G ratio) showed no discernible variation between the groups (*p*>0.05).

The serum lipid profile showed more linear (*p*<0.001) and quadratic (*p*=0.004) decreases in triglycerides in groups served energy-restricted diets with and without lysolecithin compared with groups on non-energy-restricted diets. Cholesterol significantly reduced (*p*=0.029) in C_Low_ and C_Low_E_125_ groups, which were supplied with low-energy diets alone or with 125 mg/kg lysolecithin, whereas HDL showed a linear increase (*p*=0.021), with the highest value in the C_Low_E_250_ group that fed on a low-energy diet with 250 mg/kg diet lysolecithin compared with other groups.

### 3.4 Immune and antioxidant indices

As shown in Table 6, antioxidant and lysozyme activities showed a marked decrease in MDA (*p*<0.001), with the highest level recorded in the CS group. On the other hand, SOD (*p*<0.001) and CAT (*p*<0.001) activities increased in the low-energy group and lysolecithin-treated groups compared with the control group on the standard energy level. However, TAC (*p*<0.001) increased in the lysolecithin-supplemented groups compared with the unsupplemented groups 1 and 2. IGM and LZM increased more significantly (*p*<0.001) in broilers fed low-energy feed without emulsifiers or supplemented with two levels of lysolecithin compared to those fed diets with standard energy level.

**Table 6:** Effect of the dietary supplementation of Lysolecithin to a low-energy diet on antioxidant and immunity response in broilers.

Traits	CS	C_Low_	C_Low_E_125_	C_Low_E_250_	ANOVA value	Regression	
						Linear	Quadratic
Antioxidant activities							
MDA (nmol/mL)	13.11 ± 0.62^a^	7.33 ± 0.27^b^	5.62 ± 1.24^c^	1.71 ± 0.41^d^	<0.001	<0.001	0.058
SOD (U/mL)	19.09 ± 1.09^d^	29.83 ± 0.87^c^	75.85 ± 6.29^b^	174.12 ± 5.31^a^	<0.001	<0.001	<0.001
CAT (U/mL)	2.18 ± 0.10^d^	3.22 ± 0.76^c^	6.79 ± 0.39^b^	12.83 ± 0.27^a^	<0.001	<0.001	<0.001
TAC (U/mL)	9.95 ± 0.56^c^	10.48 ± 0.04^c^	13.53 ± 0.95^b^	15.12 ± 0.93^a^	<0.001	<0.001	0.241
Immunological response							
IGM (ng/mL)	142.58 ± 12.5^d^	214.89 ± 28.9^c^	295.44 ± 17.2^b^	489.25 ± 33.7^a^	<0.001	<0.001	<0.001
LZM (ng/mL)	0.59 ± 0.16^d^	2.86 ± 0.38^c^	8.17 ± 0.54^b^	11.51 ± 1.15^a^	<0.001	<0.001	0.210

^a–d^Mean values in the same row with different superscripts differ significantly (*p*<0.05).

CS, control standard energy diet without emulsifier; C_Low_, control low-energy diet without emulsifier; C_Low_E_125_, control low-energy diet + 125 mg/kg diet lysolecithin; C_Low_E_250_, control low-energy diet + 250 mg/kg diet lysolecithin; MDA, Malondialdehyde; SOD, Superoxide dismutase; LZM, Lysozyme; CAT, Catalase; TAC, Total antioxidant capacity; IGM, Immunoglobulin M.

### 3.5 Histopathological findings

Table 7 and Figure 1 display the positive result on morphometric changes of duodenum villi of broilers from the dietary inclusion of lysolecithin in low-energy diets. VL was enhanced (*p*<0.001) in broilers fed an energy deficient regime nourished with different levels of lysolecithin contrast with that fed low density diet without emulsifier, VW (*p*=0.012) and ASA (*p*<0.001) were pointedly increased in lysolecithin supplemented groups compared with C_Low_ group. However, no significant changes were observed in CD among treatments (*p*>0.05). The VL/CD ratio decreased (*p*=0.017) in energy deficient groups with or without lysolecithin and the best value was in birds fed standard energy diet. All examined sections from intestine (groups 1 to 4) showed normal morphology of columnar epithelium that lines the muscle layers, submucosa, and mucosal villi.

**Table 7:** Effect of the dietary supplementation of lysolecithin to a low-energy diet on the duodenum morphology of broilers.

Traits	CS	C_Low_	C_Low_E_125_	C_Low_E_250_	ANOVA value	Regression	
						Linear	Quadratic
VL (μm)	1790.67 ± 54.9^a^	1220.3 ± 165.6^c^	1,336 ± 29.3^bc^	1493.3 ± 42.6^b^	<0.001	0.010	<0.001
VW` (μm)	154.67 ± 18.23^a^	109.3 ± 14.84^b^	151.3 ± 16.77^a^	161.67 ± 8.50^a^	0.012	0.167	0.013
ASA (mm^2^)	0.278 ± 0.040^a^	0.123 ± 0.013^c^	0.201 ± 0.018^b^	0.241 ± 0.012^ab^	<0.001	0.463	<0.001
CD (μm)	205 ± 30.41	172.3 ± 19.01	189.67 ± 15.89	212.67 ± 12.58	0.167	0.514	0.047
VL/CD Ratio	8.70 ± 1.05^a^	6.97 ± 0.40^b^	7.07 ± 0.42^b^	7.03 ± 0.22^b^	0.017	0.010	0.040

^a–c^Mean values in the same row with different superscripts differ significantly (*p*<0.05).

CS, control standard energy diet without emulsifier; C_Low_, control low-energy diet without emulsifier; C_Low_E_125_, control low-energy diet + 125 mg/kg diet lysolecithin; C_Low_E_250_, control low-energy diet + 250 mg/kg diet lysolecithin; VL, Villus length; VW, Villus width; ASA, Absorption surface area; CD, Crypt depth.

**Figure 1:**
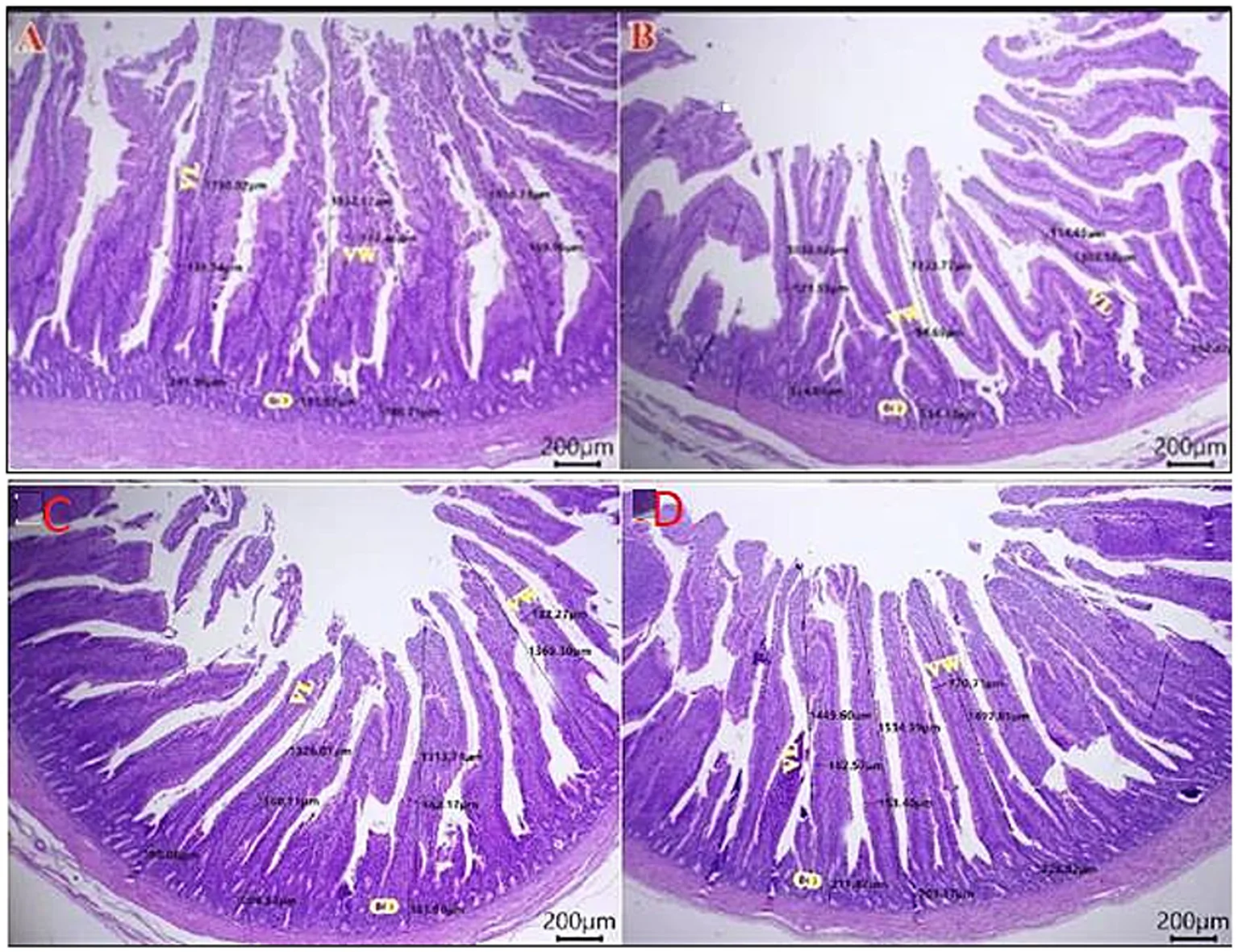
Photomicrograph of H&E-stained sections of duodenum of chickens (scale bar 200 μm) showing normal morphology of columnar epithelial lining, mucosal villi, submucosa, and muscular layers in all examined groups: **(A)** CS, control standard-energy diet without emulsifier; **(B)** CLow, control low-energy diet without emulsifier; **(C)** CLowE125, control low-energy diet + 125 mg/kg diet lysolecithin; and **(D)** CLowE250, control low-energy diet + 250 mg/kg diet lysolecithin. Higher values of intestinal parameters (villus length, villus width, and crypt depth) were at CS **(A)** and C_Low_E_250_ **(D)**. Least values were at C_Low_ **(B)**. Moderate values of intestinal parameters were in C_Low_E_125_ **(C)**.

The highest values of the intestinal morphological parameters, including villus width (VW), villus length (VL), crypt depth (CD), and absorptive surface area (ASA), were observed in Group 1 (Figure 1A), which received the standard-energy diet, followed by Group 4 (Figure 1D), which was fed the low-energy diet supplemented with a high level of lysolecithin emulsifier. In contrast, the lowest values were recorded in Group 2 (Figure 1B), which received the low-energy diet without emulsifier supplementation. Intermediate values of these intestinal morphological parameters were observed in Group 3 (Figure 1C).

### 3.6 Lysolecithin reverses the suppression of intestinal lipid-metabolism genes caused by energy restriction

The relative mRNA expression of ten genes that govern intestinal fatty-acid uptake, intracellular trafficking, hepatic re-esterification and lipoprotein assembly was quantified in birds from the four dietary groups; values normalized to the standard-energy control (CS = 1.00) are summarized in Figures 2A–C. Compared with the standard-energy control, the low-energy diet without emulsifiers (C_Low_) significantly down-regulated nine of the ten genes (*p* < 0.05). The suppression was most pronounced for the fatty-acid translocase *CD36* and the cholesterol transporter *NPC1L1* (both reduced to 0.60 of CS), the fatty-acid transporter *SLC27A4* (0.63), and the fatty-acid-binding protein *FABP* (0.67) and was more moderate for *PPARγ* (0.79), *MTTP* (0.82), *ACSL* and *MGAT2* (0.87), and *APOB* (0.89). *PPARα* was the sole exception: rather than being suppressed, it was markedly elevated in C_Low_ birds (1.78-fold of CS) (Figures 2A,B).

**Figure 2:**
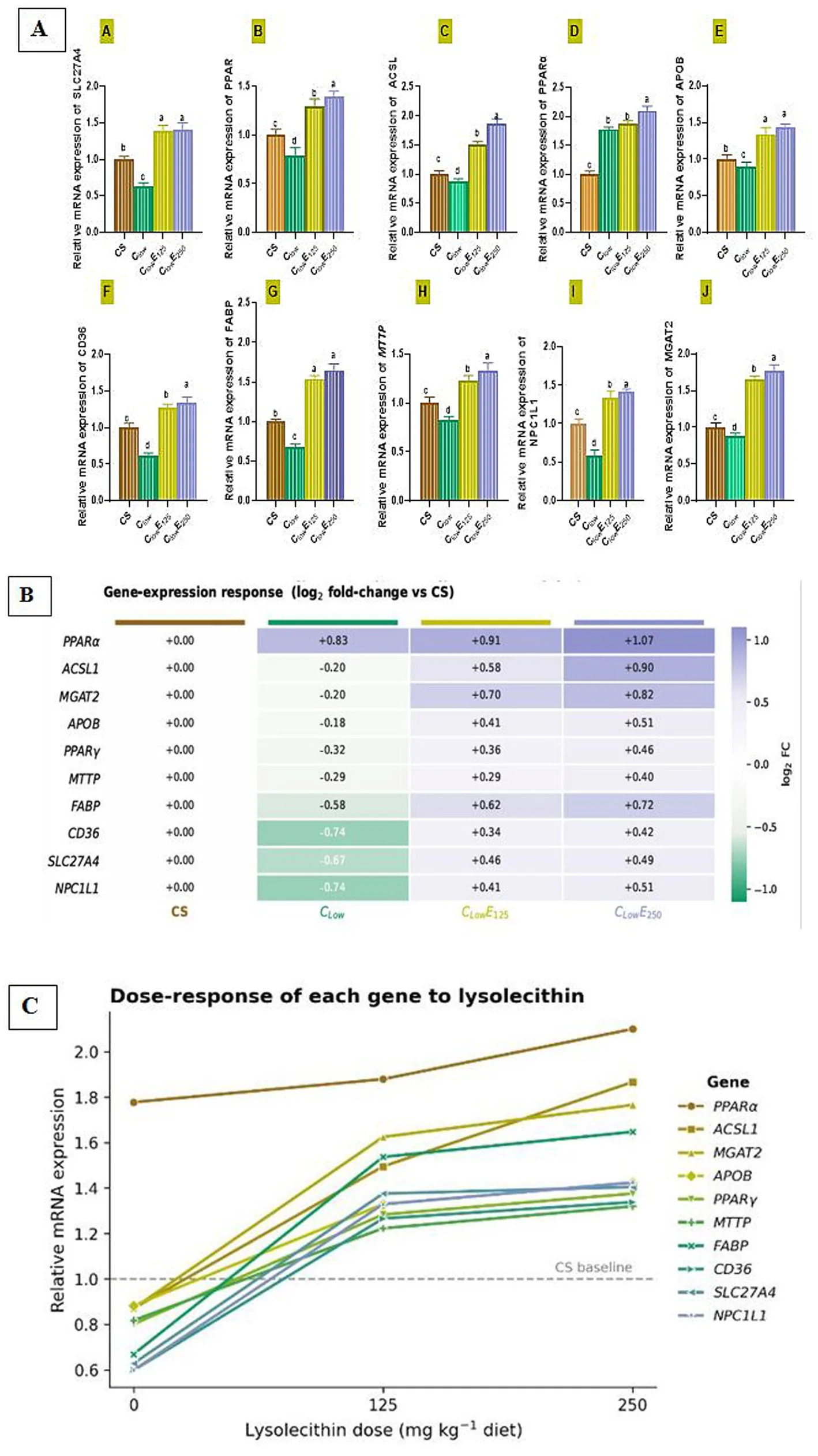
**(A)** Quantitative mRNA expression of intestinal and hepatic lipid transportation and metabolism gene, **(B)** lysolecithin remodels intestinal and hepatic lipid transportation and metabolism gene expression in heat map, and **(C)** dose–response of each gene to lysolecithin: solute carrier family 27 member 4 (*SLC27A4*); peroxisome proliferator-activated receptors alpha and gamma (*PPARα, PPARγ*); long-chain acyl-CoA synthetase family member 1 (*ACSL1*); cluster of differentiation 36 (*CD36*); Niemann-Pick C1-Like 1 (*NPC1L1*); monoacylglycerol acyltransferase 2 (*MGAT2*); microsomal triglyceride transfer protein (*MTTP*); polipoprotein B (*APOB*) and fatty acid binding protein (*FABP*); lyceraldehyde-3-phosphate dehydrogenase (*GAPDH*); *TBP*: TATA-binding protein; and *rpoD* RNA polymerase sigma factor. CS, control standard diet, C_Low_, low-energy diet, C_Low_E_125/250_, low-energy diet + 125/250 mg kg^-1^ lysolecithin.

Lysolecithin supplementation reversed this suppression and raised expression above control values for every gene. At 125 mg/kg (C_Low_E_125_), all ten genes were significantly higher than in C_Low_ birds (*p* < 0.05), and most exceeded the CS level. Increasing the dose to 250 mg/kg (C_Low_E_250_) produced a further significant increase for *PPAR*, *ACSL*, *PPARα*, *CD36*, *MTTP*, *NPC1L1*, and *MGAT2*, whereas *SLC27A4* and *FABP* reached similar values at both doses. The highest expression at 250 mg/kg was recorded for *PPARα* (2.10), *ACSL* (1.87), and *MGAT2* (1.76). The dose–response relationship is shown explicitly in Figure 2C: expression rose monotonically from the un-supplemented low-energy diet to 250 mg/kg lysolecithin, with the steepest gains for *ACSL* and *MGAT2* and a near-plateau between the two doses for *SLC27A4*, *FABP*, and *PPAR*.

### 3.7 Lysolecithin restores beneficial gut bacteria depleted by energy restriction

Culturable counts of six intestinal bacterial groups (log₁₀ CFU) are summarized in Figures 3A,B; the color scale of the source heat map spanned 2.1 to 6.1 log₁₀ CFU. Compared with CS, the low-energy diet reduced the counts of *Lactobacillus* and *Bacillus* most strongly (each falling by roughly 1.2–1.4 log₁₀ CFU) and also lowered *Firmicutes* (by about 1 log₁₀ CFU) and *Bifidobacterium*, while *Bacteroides* increased (by about 0.9 log₁₀ CFU) and *Enterobacteriaceae* changed little. Lysolecithin supplementation progressively reversed these shifts: at 125 and 250 mg/kg, *Lactobacillus*, *Bacillus*, and *Bifidobacterium* recovered to values close to or above the CS level, and *Firmicutes* increased partially toward control. *Bacteroides* remained elevated above CS in all three low-energy groups irrespective of supplementation, and *Enterobacteriaceae* stayed near the control value throughout.

**Figure 3:**
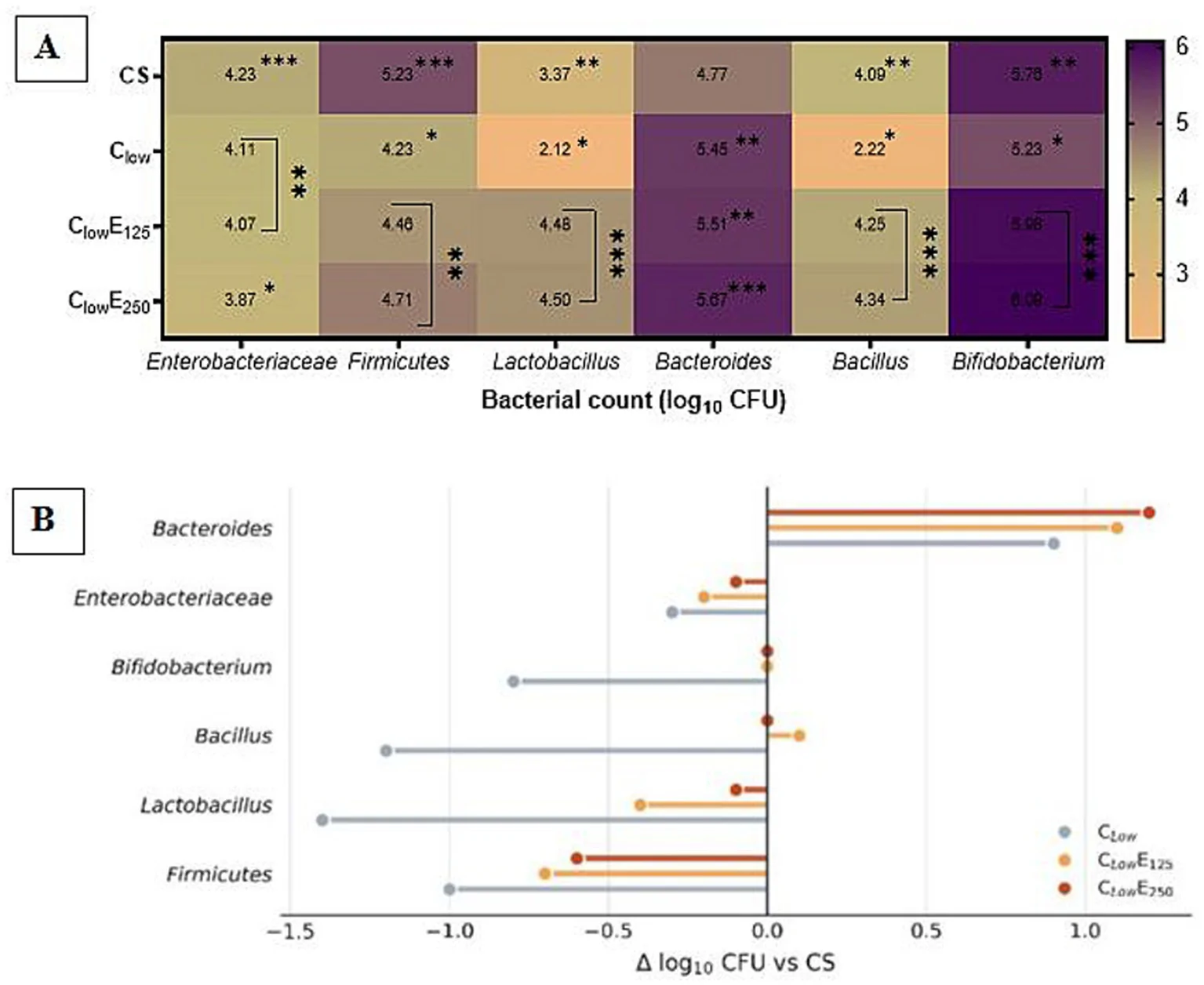
**(A)** Quantitative DNA copies of key bacterial groups, including *Enterobacteriaceae, Firmicutes*, *Lactobacillus*, *Bacteroides*, *Bacillus* spp., and *Bifidobacterium*, expressed as log_10_ CFU/g of sample in heatmap, and **(B)** illustration of the gut microbiota shift relative to CS group. Asterisks denote a statistically significant difference from the CS (standard-energy control) group (*p < 0.05). CS: control standard diet; C_Low_: low-energy diet; C_Low_E_125/250_: Low-energy diet + 125/250 mg kg^−1^ lysolecithin.

### 3.8 Economic efficiency

The effect of lysolecithin supplementation to the low-energy diet on the economic performance of broilers is presented in Table 8. The total feed cost, fixed cost, and total cost did not differ significantly among the dietary groups (*p* > 0.05). Feed cost per kilogram of body weight gain was significantly affected by treatment (*p* = 0.033); the lowest value was recorded in the CL_ow_E_250_ group, which differed significantly from the other groups and showed a significant quadratic response to lysolecithin level (*p* = 0.016).

**Table 8:** Effect of the dietary supplementation of lysolecithin to a low-energy diet on the economic efficiency of broilers.

Traits	CS	C_Low_	C_Low_E_125_	C_Low_E_250_	ANOVA value	Regression	
						Linear	Quadratic
Feed cost/kg gain ($)	0.571 ± 0.015^a^	0.592 ± 0.005^a^	0.610 ± 0.013^a^	0.556 ± 0.005^b^	0.033	0.103	0.016
Total feed cost ($)	1.257 ± 0.023^a^	1.224 ± 0.010^a^	1.260 ± 0.024^a^	1.225 ± 0.006^a^	0.353	0.968	0.103
Total cost ($)	1.287 ± 0.023^a^	1.254 ± 0.010^a^	1.290 ± 0.024^a^	1.255 ± 0.006^a^	0.353	0.968	0.103
Total revenue ($)	3.372 ± 0.033^a^	3.168 ± 0.002^b^	3.169 ± 0.010^b^	3.371 ± 0.014^a^	<0.001	0.003	<0.001
Net revenue ($)	2.085 ± 0.052^a^	1.913 ± 0.008^b^	1.878 ± 0.032^b^	2.116 ± 0.019^a^	0.002	0.018	0.002
Economic efficiency	1.622 ± 0.068^a^	1.526 ± 0.019^a^	1.457 ± 0.051^b^	1.686 ± 0.023^a^	0.030	0.082	0.012
Profitability index	0.618 ± 0.010^a^	0.604 ± 0.003^a^	0.593 ± 0.008^b^	0.628 ± 0.003^a^	0.028	0.099	0.013
Benefit–cost ratio	2.622 ± 0.068^a^	2.526 ± 0.019^a^	2.457 ± 0.051^b^	2.686 ± 0.023^a^	0.030	0.082	0.012

^a, b^Mean values in the same row with different superscripts differ significantly (*p* < 0.05).

CS, control standard energy diet without emulsifier; C_Low_, control low-energy diet without emulsifier; C_Low_E_125_, control low-energy diet + 125 mg/kg diet lysolecithin; C_Low_E_250_, control low-energy diet + 250 mg/kg diet lysolecithin.

Total revenue and net revenue differed markedly among groups (*p* < 0.001 and *p* = 0.002, respectively). Birds fed the standard-energy diet (CS) and those fed the low-energy diet supplemented with 250 mg/kg lysolecithin (CL_ow_E_250_) achieved the highest total and net revenues, whereas the lowest values were recorded in the unsupplemented low-energy group (C_Low_) and the C_Low_E_125_ group.

Economic efficiency, profitability index, and the benefit–cost ratio followed the same pattern, differing significantly among treatments (*p* = 0.030, 0.028, and 0.030, respectively). Supplementation with 250 mg/kg lysolecithin restored these indices to values statistically comparable to those of the standard-energy control (CS) and superior to those of the unsupplemented low-energy diet, with the C_Low_E_250_ group recording the highest benefit–cost ratio and economic efficiency. All three indices exhibited a significant quadratic response to dietary lysolecithin level (*p* ≤ 0.013), reflecting the decline at 125 mg/kg and the subsequent improvement at 250 mg/kg.

## 4 Discussion

Lowering the dietary metabolizable energy density of broiler diets reduces feed cost, which is the single largest expense in poultry production, but the resulting energy deficit depresses growth because young birds have a limited capacity to digest and absorb dietary fat; supplementing such low-energy diets with lysolecithin counteracts this limitation, since its potent emulsifying action increases the active surface area of lipid droplets, promotes micelle formation, and thereby enhances fat digestion and energy utilization to restore performance (39, 40). The present study was uniquely designed to evaluate, for the first time, the efficacy of two doses of hydrolyzed lecithin (125 and 250 g/t) in broiler diets reformulated with an 80 kcal/kg reduction in metabolizable energy, a dose-by form by energy combination not previously reported, with specific emphasis on its modulation of the cecal microbial community and the expression of key lipid-metabolism genes. In this study, broilers on the standard-energy diet and those on the low-energy diet with high-dose hydrolyzed lecithin performed best, showing the highest final body weight and weight gain and the most favorable FCR, whereas the low-energy-only group (C_Low_) had the poorest growth and worst FCR. Supplementing the low-energy diet with lysolecithin at 125 and 250 mg/kg significantly increased body weight and weight gain while lowering FCR (improved feed efficiency), with 250 mg/kg being the most effective, restoring performance close to that of the basal diet. Feed intake remained largely unchanged across all groups. That improvement was largely due to better nutrient utilization rather than higher feed consumption.

These findings show that adding exogenous emulsifiers to broilers’ diets deficient in calories can enhance fat digestion and energy availability, which in turn can partially increase growth. Our results match with results from other studies, (41) where it was demonstrated that birds fed reduced-energy regimes containing lysophospholipid at addition levels of 0.10 and 0.15% had greater body weight. Another study (42) reported that when broilers were fed lysolecithin at 25–50 g/kg at 21 days of age and 50 g/kg at 35 days of age, they acquired more body weight, and feed efficiency increased with 50 g/kg diet supplementation level. There have been prior reports of growth performance increases in broiler diets after lysophospholipid-based products were added (23, 43–46). Supplementation dosages should be advised based on dietary energy levels after finding that the lower-most amount of lysolecithin, 250 g/t, out of all evaluated doses (apart from 500 and 750 g/t), improved growing performance when applied to lower-energy regimes (47). The performance outcomes obtained are consistent with other studies (16, 23, 43) who demonstrated that lysophospholipid supplementation significantly impacted the growth performance metric. In addition, this study agreed with those (14, 48) that found that A 0.05% lysolecithin dietary supplement can enhance growth performance. In broiler diets reformulated to a lower metabolizable energy (ME) density, the resulting energy gap typically depresses body weight gain and worsens feed conversion, particularly in young birds whose endogenous bile salt secretion and lipase activity are not yet sufficient to efficiently emulsify and hydrolyze dietary fat (49). Hydrophilic lysophospholipid has a markedly higher hydrophilic–lipophilic ratio that can lower interfacial tension at the oil–water interface, it disperses dietary lipids into finer droplets, expands the active surface area available for pancreatic lipase, and accelerates the incorporation of free fatty acids and monoglycerides into mixed micelles, thereby promoting their transport across the unstirred water layer to the enterocyte brush border (23). This enhanced micellar solubilization improves the apparent digestibility of fat and gross energy, allowing birds to extract more usable energy from a diet of reduced caloric density and partially or fully offsetting the deficit. The improved energy and nutrient supply is reflected in heavier final body weights and improved feed conversion ratios reported when lysolecithin is added to low-energy broiler diets (23, 49), and at the molecular level it has been linked to up-regulation of intestinal nutrient transporter and growth related genes, indicating that the performance response reflects both improved luminal lipid handling and enhanced absorptive capacity rather than emulsification alone (14).

Supplementing low-energy broiler diets with lysolecithin had no effect on carcass yield or liver percentage, while heart and gizzard percentages showed a slight improvement with lysolecithin addition. In accordance, there was no discernible change in carcass yield in earlier research using pure lysolecithin (47, 50, 51). Other studies (52, 53) discovered that adding an emulsifier had no effect on breast yield, abdominal fat, or carcass characteristics. Overall, the present findings indicate that lysolecithin can partly counteract the growth and carcass limitations associated with low-energy diets by improving lipid utilization without adversely affecting internal organ development. When broilers were fed a low-energy regime accompanied with 125 and 250 mg lysolecithin/kg diet, their ALT activity decreased, indicating improved liver function and less hepatocellular stress when compared with low-energy diet. One important measure of liver health is ALT, and a decrease in it typically indicates improved hepatic lipid metabolism and less fat buildup in hepatocytes. The safety of inclusion at this dosage is confirmed by the unchanged levels of AST, albumin, globulin, and total protein, which show that supplementing lysolecithin did not cause liver damage or change protein-production pathways. This is consistent with the outcomes of a study (12) that found increasing micelle production and nutrient availability enhanced liver enzymatic performance and facilitated lipid digestion and absorption. Additionally, lysolecithin addition had no discernible effect on total protein, albumin, or globulin concentrations and albumin globulin ratio.

In the present study, dietary lysolecithin reduced serum levels of TG and cholesterol and increased HDL, which is in line with prior research that established the ability of lysolecithin to lower TG and cholesterol while improving HDL (54). Moreover, emulsifiers improve intestinal absorption and reduce circulating cholesterol/triglyceride concentration (13). our results match with (55) who found that the lipid profile was enhanced by dietary lysophospholipids. Lysophospholipid supplementation also raised HDL while lowering serum TG, total cholesterol, and LDL (56). Other studies (41, 57) demonstrated that nourishment of reduced-energy diets with lysophospholipid increases HDL and occasionally decreases TG or LDL, also lysolecithin reduce serum TC and LDL levels, but increase HDL and triglyceride levels of broilers (58). The reduction in uric acid and serum creatinine concentrations in broilers fed a low-energy food accompanied with lysolecithin when compared with low energy diet control indicated an optimizing protein metabolism efficiency. Matching with our findings (54), one study reported that administering lecithin plus an emulsifier improved kidney biochemical markers by lowering serum urea and uric acid. The findings demonstrated that lysolecithin inclusion raised the activities of TAC, SOD, and CAT enzymes while decreasing the amount of MDA in the serum in contrast to those given standard and deficient energy diets without emulsifier. The observed antioxidant performance of emulsifiers may be attributed to their distinct molecular structure (54). As strong lipid-soluble antioxidants, phospholipids can guard against oxidative stress and damage from free radicals, because they include amino groups and choline. Furthermore, the antioxidant properties of lysolecithin are attributed to its capacity to promote oxidative resistance, lessen liver damage, and improve the conflict to oxidation of oils and fats through the phospholipid components’ defensive action (59). Our outcomes agree with (60) a study that observed higher TAC, GPX, CAT, and SOD activities and lowered MDA levels, which demonstrate the LPL-improving action via causing antioxidant-oxidative equilibrium. Moreover, lysophospholipid emulsifiers (especially at 0.5 g/kg diet) improved the antioxidant capability by enhancing the antioxidant enzyme actions of SOD, CAT, and GPX, as well as the phagocytic action (55). Our results match with a study (39) who noticed that adding lysolecithin to the meal improved SOD and GPX activity while lowering MDA.

Lysolecithin supplementation in low-energy diets improved the broilers’ immunological response in the current investigation, as shown by elevated lysozyme activity and serum IgM concentration. These effects are in line with earlier investigations showing that emulsifiers like lecithin or lysolecithin can boost innate and humoral immunity in broilers by improving food absorption and lipid digestion, which in turn supports immune cell function (61, 62). Also, adding lysolecithin to low-energy diets lowered metabolic stress and enhanced immunological markers in general (49). One important metric for evaluating gut health is the intestinal mucosa’s morphology. The current study found that a low-energy regimen considerably reduced the height of the duodenal villus and the absorptive surface area in contrast to the usual diet control group. This suggested that energy restriction below requirements can impair the normal intestinal mucosa’s structural integrity. However, these morphological characteristics were partially or significantly restored in a dose-dependent method by dietary supplementation with lysolecithin at both 125 and 250 mg/kg diet. Villus height increased from approximately 1,222 μm in the unsupplemented low-energy group to approximately 1,493 μm at the maximum dose (250 mg/kg diet), which was close to the level observed in the standard energy diet (1,792 μm). Likewise, villus width and absorptive surface area were enhanced. Additionally, crypt depth tended to rise in treated broilers, most likely due to increased cell proliferation supporting villus renewal. Emulsifiers improve intestinal morphology; however the exact mechanism of action is still unclear. By decreasing intestinal fermentation and so minimizing villus surface damage in broilers, the emulsifier supplement may enhance the structure of the intestinal mucosa by inducing micelle formation in the gut (7). The proposed mechanism is that lysolecithin promotes micelle formation and improves lipid presentation at the brush border, supplying enterocytes with the energy and substrate needed for proliferation and villus elongation; this is reflected in increased villus height and an improved villus height–to–crypt depth (VH: CD) ratio, the latter signaling greater epithelial maturity and reduced cell-turnover demand (20). Moreover, the addition of lysolecithin increased cell mitosis in the broiler chickens’ duodenum region, stimulating an increase in villus height. This suggests that the emulsifier worked by encouraging the formation of the villi by reducing and stabilizing the size of micelles to be absorbed; emulsification might thereby encourage division and renewal of cells in the villus, increasing its height (63). The avoidance of cell destruction and the elevation of gene appearance linked to enhancing intestinal morphology in broilers may be the reasons for LL’s positive impact on intestinal development (64, 65). Also, better nutritional absorption is associated with a higher VH/CD ratio (66). Additionally, our findings match with studies (67, 68) who observed an improvement in duodenum villus height alongside a reduction in crypt depth of duodenum in broilers fed lysophospholipid diets. The results also align with researchers (8) who showed that broilers fed low metabolizable energy and/or low crude protein diets showed improvements in duodenal villus height and villus height/crypt depth when administered a multicomponent emulsifier blend. Other studies (23, 63) stated that dietetic lysolecithin raised the height of the mucosa. Also, supplementing low-energy diets with lysophospholipids resulted in a decrease in the depth of the duodenum’s crypt and an increase in the height of the jejunal villus (47, 69). Our results are in line with a study (67) that found that broilers fed diets supplemented with lysophospholipid showed increase in villus height while the duodenum’s crypt depth decreases. Taken together, the low-energy literature suggests that lysolecithin’s histological benefit is largely a function of relieving the substrate and emulsification limitation imposed by energy reduction. This was precisely the aim of the present study, although the magnitude of response still varies with dose, energy gap, and intestinal segment.

In modern broiler production, the bulk of dietary energy is supplied by added fat, yet younger birds emulsify and absorb lipid inefficiently as their endogenous bile-salt and pancreatic-lipase systems are not fully developed. Lysolecithin, a lysophospholipid emulsifier obtained by phospholipase hydrolysis of soybean lecithin, is widely included in poultry feed to compensate for this limitation. In the present study, lowering dietary energy without an emulsifier (C_Low_) consistently depressed the intestinal expression of genes governing fatty-acid uptake, intracellular trafficking, triacylglycerol re-esterification, and lipoprotein assembly relative to the standard-energy control, whereas graded inclusion of lysolecithin at 125 and 250 mg/kg not only reversed this suppression but raised the expression of nearly every gene above the CS level. Taken together, these patterns indicate that lysolecithin acts on the broiler intestine at the transcriptional level, beyond its classical role as a surface-active agent in agreement with reports that lysolecithin upregulates nutrient-transporter-related genes independently of dietary energy density (14) and that emulsifier supplementation reshapes the intestinal lipid-handling transcriptome of broilers (13, 70). The downregulation observed in C_Low_ birds is biologically coherent. Expression of the apical fatty-acid transporters and of the FABP–MTTP–APOB module is substrate-responsive: when dietary fat and, therefore, the luminal load of long-chain fatty acids and monoacylglycerols is reduced, the enterocyte has less need to invest in lipid-import and chylomicron-assembly proteins. A comparable feed-conversion penalty and depressed nutrient utilization in low-energy broiler diets has been documented previously (46, 71, 72), and the present gene-expression data provide a mechanistic correlate, showing that the energy-reduced diet reduces the transcriptional program that supports lipid absorption. This makes the rescue achieved by lysolecithin all the more notable, because expression was restored even though the diet itself remained energy-reduced.

Three of the genes examined—SLC27A4, CD36, and FABP—functioned at the first step of lipid absorption: the movement of long-chain fatty acids from the mixed micelle into the enterocyte and their subsequent cytosolic trafficking. CD36 (fatty-acid translocase) is a brush-border scavenger receptor that binds long-chain fatty acids with high affinity and directs them toward esterification and prechylomicron formation; SLC27A4 (fatty-acid transport protein 4) is the principal fatty-acid transport protein of the apical enterocyte membrane (73); and FABP shuttles absorbed fatty acids through the cytosol to the endoplasmic reticulum, where, together with CD36 and the microsomal transfer protein, it participates in budding of the prechylomicron transport vesicle (74). In the present study, all three genes were depressed by energy reduction and then strongly upregulated by lysolecithin *SLC27A4* and *FABP* reaching a plateau already at 125 mg/kg, while *CD36* rose dose-dependently to a maximum at 250 mg/kg. This mirrors the emulsifier-driven increase in intestinal *CD36*, *SLC27A4*, and *FABP* reported in yellow-feathered broilers (13) and the upregulation of jejunal fatty-acid transporters by lysolecithin (14, 70). Functionally, this implies a greater fatty-acid uptake capacity per unit of intestinal tissue, enabling the bird to extract more energy from a diet that contains less of it.

Once inside the enterocyte, fatty acids must be activated to acyl-CoA thioesters before they can be esterified—a reaction catalyzed by the long-chain acyl-CoA synthetase (ACSL) family; the dominant intestinal isoform contributes the large majority of mucosal ACSL activity and is rate-influencing for dietary fat absorption (75). *ACSL* displayed the steepest dose–response of any gene in the dataset, climbing from a depressed value in C_Low_ to roughly twice the CS level at 250 mg/kg. Because acyl-CoA formation sustains the inward concentration gradient that drives continued fatty-acid uptake and supplies substrate for triacylglycerol synthesis, the marked, dose-dependent induction of *ACSL* by lysolecithin provides a metabolic link between the enhanced uptake step (*CD36, SLC27A4,* and *FABP*) and the downstream re-esterification and packaging steps.

*MGAT2* (monoacylglycerol acyltransferase 2) catalyzed the first acylation of the monoacylglycerol pathway, which accounts for the majority of triacylglycerol re-synthesis in the enterocyte endoplasmic reticulum (75). Its expression followed the same dose-dependent pattern, being suppressed in C_Low_ and rising to approximately 1.8-fold of CS at the higher lysolecithin dose. Upregulation of *MGAT2* indicates that lysolecithin not only increases the entry of fatty acids but also expands the capacity to re-esterify them into triacylglycerol, the neutral-lipid cargo of chylomicrons undergoing a coordinated response that would be expected to raise the throughput of the absorptive pathway as a whole. The assembly and secretion of chylomicrons depend on two obligatory components: *MTTP* and *APOB. APOB* is the non-exchangeable structural scaffold of the nascent particle, whereas *MTTP* both stabilizes the elongating APOB polypeptide and transfers triacylglycerol, phospholipid, and cholesteryl ester onto it; loss of either function abolishes chylomicron formation (76). Both genes were depressed by energy reduction and restored above the CS level by lysolecithin *APOB* plateauing at 125 mg/kg and *MTTP* increasing dose-dependently. Coordinate induction of intestinal *MTTP* and *APOB* by a dietary emulsifier has likewise been reported in broilers (13), and the parallel behavior of the two genes here is consistent with their shared, frequently co-regulated role in lipoprotein output. By upregulating this export module alongside the uptake and re-esterification genes, lysolecithin appears to strengthen every sequential stage of the trans-enterocyte lipid pathway.

*NPC1L1* (Niemann-Pick C1-Like 1) is the apical sterol transporter that mediates intestinal absorption of cholesterol and certain fat-soluble vitamins and is the molecular target of the cholesterol-absorption inhibitor ezetimibe (77). *NPC1L1* was the most strongly suppressed gene in the C_Low_ group and was robustly restored by lysolecithin in a dose-dependent manner. Because micellar solubilization is a prerequisite for *NPC1L1-*mediated sterol uptake, the improved emulsification provided by lysolecithin would be expected to increase the delivery of cholesterol to the transporter; the concurrent transcriptional upregulation points to an additional, expression-level effect. This indicated that the benefit of lysolecithin extends beyond triacylglycerol to the absorption of sterols and, by extension, of lipophilic micronutrients.

The two nuclear-receptor genes, *PPARγ* and *PPARα*, offer a plausible mechanistic explanation for this broad transcriptional response. PPARα is abundantly expressed in enterocytes, where it acts as a lipid-activated transcription factor governing fatty-acid transport, oxidation, and lipoprotein-related genes (78). Notably, *PPARα* was the only gene upregulated by energy reduction itself, with C_Low_ birds expressing it at about 1.8-fold of CS. This is consistent with the established role of PPARα as a sensor of negative energy balance: energy or fat restriction mimics a fasting state, in which PPARα is induced to maximize oxidation of the limited fatty-acid supply (79). Lysolecithin raised *PPARα* further in a dose-dependent fashion and increased the second *PPAR* gene from a depressed C_Low_ value to above CS. Two mechanisms can account for this. First, the greater flux of absorbed fatty acids generated by lysolecithin supplies endogenous PPAR ligands, since long-chain fatty acids and their acyl-CoA derivatives are natural PPAR agonists. Second, lysophosphatidylcholine, the principal active species in lysolecithin, has itself been shown to act as a signaling lipid capable of activating PPAR-class receptors (80). Either route would establish a feed-forward loop in which lysolecithin both delivers more substrate and amplifies the transcriptional programme that handles it, explaining why *CD36*, *ACSL*, *MTTP*, *MGAT2*, and the remaining PPAR-responsive genes rose in concert. Increased intestinal and hepatic *Pparα* expression following emulsifier supplementation has been reported previously in broilers (13), which supports this interpretation.

A consistent feature of the results is the graded response to lysolecithin dose. For *PPAR*, *ACSL*, *CD36*, *MTTP*, *NPC1L1*, *MGAT2* and *PPARα*, 250 mg/kg produced significantly higher expression than 125 mg/kg, whereas *SLC27A4*, *APOB*, and *FABP* had already reached their maximum at the lower inclusion. This suggests that the apical fatty-acid-uptake and apoB-scaffold steps saturate at a relatively modest emulsifier level, while the activation, re-esterification, sterol-uptake, and regulatory steps continue to respond up to 250 mg/kg. From an applied standpoint, the persistence of a dose effect at 250 mg/kg implies that the higher inclusion confers additional benefit for lipid utilization in energy-reduced broiler diets, although accompanying performance and economic data would be required to define the optimum.

Overall, reducing dietary energy without an emulsifier downregulated the intestinal genes responsible for fatty-acid uptake (*CD36*, *SLC27A4*, and *FABP*), fatty-acid activation (*ACSL*), triacylglycerol re-synthesis (*MGAT2*), lipoprotein assembly (*MTTP* and *APOB*), and cholesterol absorption (*NPC1L1*), while upregulating the energy-sensing receptor *PPARα*. Supplementing the low-energy diet with lysolecithin reversed these changes and, for most genes, raised expression above the standard-energy control in a dose-dependent manner. The coordinated induction of every stage of the trans-enterocyte lipid pathway, together with the upregulation of *PPAR* and *PPARα*, indicates that lysolecithin improves lipid utilization in broilers through a combination of its emulsifying action and a genuine nutrigenomic effect on the intestinal lipid-metabolism transcriptome, allowing birds fed energy-reduced diets to absorb dietary lipid more efficiently.

The gut microbiota of broiler chickens is a key determinant of nutrient utilization, intestinal development, and resistance to enteric pathogens, and its composition is highly responsive to diet (81, 82). In the present study, reducing dietary energy without an emulsifier shifted the intestinal bacterial community, lowering the counts of *Lactobacillus*, *Bacillus*, *Bifidobacterium*, and *Firmicutes* while raising *Bacteroides*, whereas supplementing the low-energy diet with lysolecithin restored the depleted beneficial genera. These observations indicate that lysolecithin does more than aid fat digestion: it also helps preserve a favorable microbial balance when dietary energy is reduced.

The microbial shift caused by the supplemented low-energy diet is consistent with the well-documented sensitivity of the broiler cecal community to dietary lipid and energy supply. Dietary fat source and concentration significantly shape the broiler cecal microbiome, in which *Firmicutes* and *Bacteroidetes* form the dominant and most diet-responsive phyla (83), and cecal microbial composition is closely linked to the bird’s nutrient and energy metabolism (84). Reducing dietary energy, therefore, alters substrate availability in the gut lumen and, as observed here, can depress lipophilic and acid-tolerant genera such as *Lactobacillus* and *Bacillus* while favoring *Bacteroides*.

The central finding of this study, that lysolecithin restored and increased the Lactobacillus population, is in close agreement with previous broiler trials. Salari et al. reported that dietary lysophospholipid supplementation significantly improved the cecal *Lactobacillus* population of broiler chickens (20), and Movagharnejad et al. observed comparable modulation of the intestinal microbial population when lysophospholipid was added to low metabolizable-energy broiler diets (24), a design closely matching the present experiment. Bassareh et al. likewise found that lysophospholipid supplementation altered cecal microbiota activity in broilers (21). The most probable mechanism is indirect: by improving the micellar solubilization and emulsification of dietary fat, lysolecithin enhances lipid digestion and intestinal morphology (13, 14, 55), creating a luminal and mucosal environment that favors the proliferation of beneficial *lactobacilli*. Lysolecithin supplementation also restored *Bifidobacterium* to the level of standard-energy control. Together with *Lactobacillus*, *Bifidobacterium* is widely regarded as a beneficial member of the poultry gut community that contributes to colonization resistance and carbohydrate fermentation; its recovery alongside *Lactobacillus* points to a coordinated improvement in gut health rather than an isolated effect on a single taxon, in line with reports that Lys phospholipid- and emulsifier-based interventions improve the overall intestinal environment of broilers (13, 55).

The recovery of *Bacillus* counts under lysolecithin supplementation is a further favorable outcome. *Bacillus* species are increasingly valued as beneficial feed additives in broilers: *Bacillus subtilis* has been shown to shift the broiler gut microbiome toward butyrate-producing bacteria and to improve intestinal histomorphology and growth performance (22, 85). The restoration of this genus therefore reinforces the interpretation that lysolecithin supports, rather than disturbs, the beneficial component of the broiler microbiota.

*Bacteroides* remained elevated in all low-energy groups, including the lysolecithin-supplemented ones. *Bacteroidetes* are major carbohydrate- and fiber-degrading members of the broiler cecal community and contribute to host energy harvest and short-chain fatty acid production (84); their persistence is consistent with the strong influence of dietary energy and substrate composition on this phylum and does not in itself indicate an adverse effect. Notably, *Enterobacteriaceae* that includes potential enteric pathogens remained essentially unchanged across all treatments, showing that lysolecithin enriched beneficial genera without promoting potentially harmful ones. Taken together, these results show that supplementing a low-energy broiler diet with lysolecithin partially corrects the dysbiosis induced by energy restriction, restoring beneficial *Lactobacillus*, *Bifidobacterium*, and *Bacillus* populations while leaving *Enterobacteriaceae* unaffected.

Feed represents the single largest variable cost in broiler production, so any nutritional strategy that lowers dietary energy without depressing performance has a direct bearing on profitability (20). In the present study, reducing the dietary metabolizable energy by 80 kcal/kg lowered the total feed and production costs, but the unsupplemented low-energy diet (C_Low_) also depressed total and net revenue, indicating that the cost saving alone did not translate into an economic advantage. Supplementing the low-energy diet with lysolecithin, particularly at 250 mg/kg (C_Low_E_250_), restored total revenue, net revenue, economic efficiency, profitability index, and benefit–cost ratio to values statistically comparable with, or numerically superior to, the standard-energy control, while feed intake and total feed cost remained largely unchanged. This indicates that the economic gain was driven by improved nutrient and energy utilization rather than by increased feed consumption, in agreement with earlier reports that exogenous emulsifiers and lysophospholipids enhance fat digestion and feed efficiency in energy-restricted broiler diets and thereby improve production economics (71). Comparable improvements in feed efficiency and profitability following lysolecithin or combined-emulsifier supplementation of low-energy broiler diets have been documented by Saleh et al. (57), who similarly attributed the response to enhanced lipid emulsification and energy availability. Collectively, these findings demonstrate that supplementing a low-energy diet with lysolecithin at 250 mg/kg is an economically favorable strategy that offsets the performance penalty of dietary energy restriction and improves the overall profitability of broiler production. In agreement with previous broiler studies (20, 21, 24), lysolecithin acts as an emulsifier that allows dietary energy to be reduced without compromising the balance of the intestinal microbiota, supporting its practical use in low-cost broiler feed formulation.

## 5 Conclusion

Supplementing low-energy broiler diets with lysolecithin restored nutrient utilization and growth performance to levels matching or exceeding those of birds fed standard-energy diets, with higher carcass yield reflecting improved fat digestion and energy availability. Blood biochemical markers remained within normal physiological ranges, confirming the safety of supplementation, while lower MDA concentrations alongside elevated SOD, CAT, and TAC indicated reduced oxidative stress and strengthened antioxidant defense. Enhanced lysozyme activity and IgM levels point to improved immune competence, likely mediated by better lipid absorption and stabilization of immune-cell membranes, and the greater villus length and villus-to-crypt-depth ratio further denote improved intestinal integrity and absorptive capacity. At the microbial and molecular level, lysolecithin shifted the cecal community toward beneficial populations while up-regulating the expression of key lipid-metabolism genes, mechanistically linking enhanced fat utilization to the observed gains in performance and health. Collectively, these findings identify lysolecithin at 250 mg/kg as an effective nutritional strategy for sustaining broiler performance, health, and immunity in energy-reduced diets. By concurrently characterizing these complementary outcomes, this study offers a uniquely holistic picture of how lysolecithin improves lipid utilization and overall gut health in energy-restricted broiler diets.

## Data Availability

The data presented in this study are available in the article, and upon request from the corresponding author.
